# Supplementary material for: The causal role of circulating inflammatory markers in osteoporosis: a bidirectional Mendelian randomized study
Source: Front Immunol. 2024 Jul 18;15:1412298. doi: 10.3389/fimmu.2024.1412298 (PMC11291241; doi:10.3389/fimmu.2024.1412298)
Supplement: Supplementary file 1 [file DataSheet_1.zip › supplementary material/OSTEOPOROSIS-CIM/GCST90274822.tsv.gz_leave_one_out.pdf]

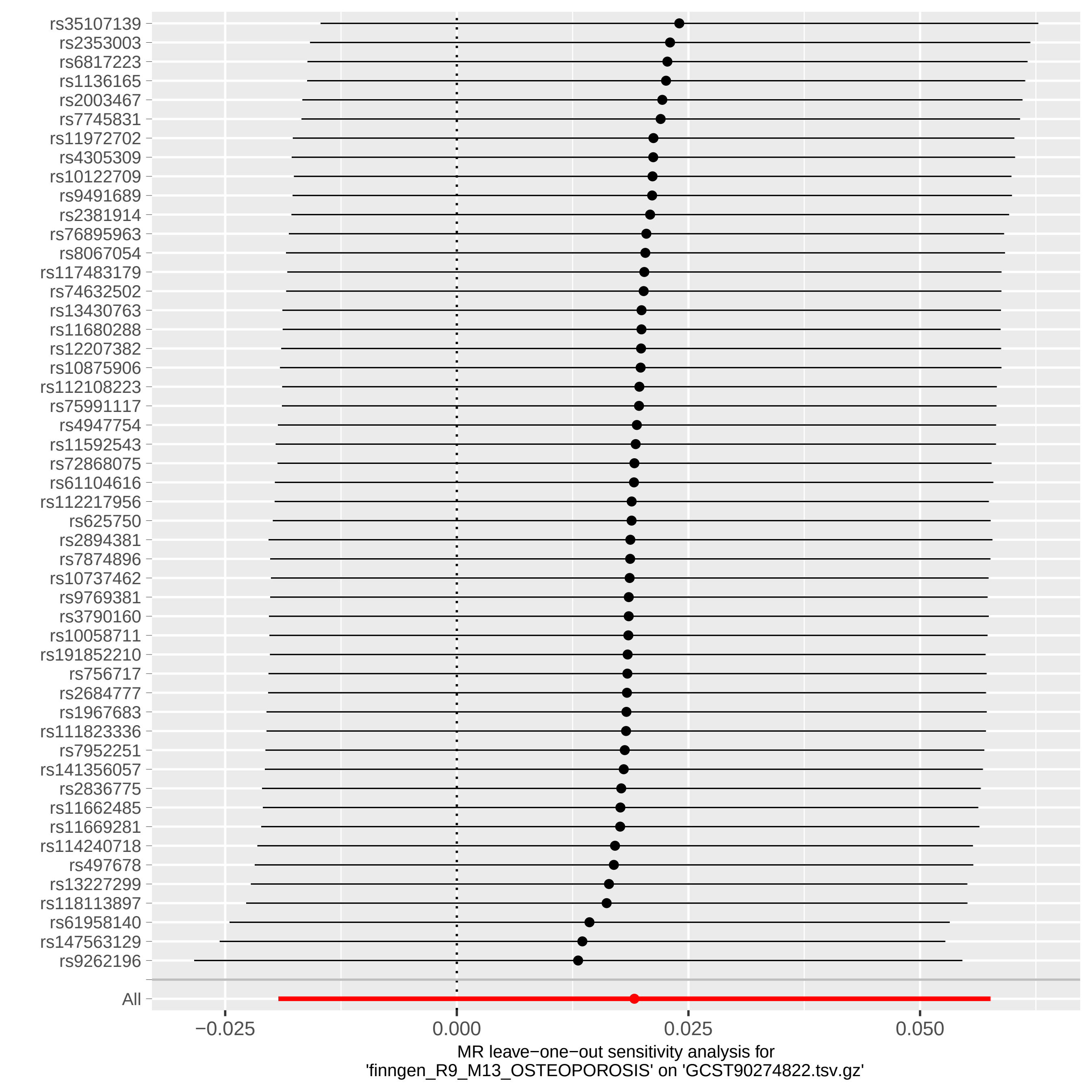

rs35107139
rs2353003
rs6817223
rs1136165
rs2003467
rs7745831
rs11972702
rs4305309
rs10122709
rs9491689
rs2381914
rs76895963
rs8067054
rs117483179
rs74632502
rs13430763
rs11680288
rs12207382
rs10875906
rs112108223
rs75991117
rs4947754
rs11592543
rs72868075
rs61104616
rs112217956
rs625750
rs2894381
rs7874896
rs10737462
rs9769381
rs3790160
rs10058711
rs191852210
rs756717
rs2684777
rs1967683
rs111823336
rs7952251
rs141356057
rs2836775
rs11662485
rs11669281
rs114240718
rs497678
rs13227299
rs118113897
rs61958140
rs147563129
rs9262196
All
−0.025
0.000
0.025
0.050
MR leave−one−out sensitivity analysis for
'finngen_R9_M13_OSTEOPOROSIS' on 'GCST90274822.tsv.gz'
